# Cases of Chronic Pleurisy at the Louisville Marine Hospital

**Published:** 1854-06

**Authors:** Austin Flint

**Affiliations:** Professor of the Theory and Practice of Medicine in the University of Louisville


### BUFFALO MEDICAL JOURNAL

AND

### MONTHLY REVIEW.

**VOL. 10.** JUNE, 1854. **NO. 1.**

### ORIGINAL COMMUNICATIONS.

ART. I.— *Cases of Chronic Pleurisy at the Louisville Marine Hospital.* By Austin Flint, M. D., Professor of the Theory and Practice of Medicine in the University of Louisville.

In a series of interesting and instructive cases of disease which have been presented at the Louisville Marine Hospital during the past few weeks of the present term of my service in that institution, are included several cases of *Chronic Pleurisy.* I have selected these as the subjects of this report for the following reasons:

1st. The affection is comparatively rare, but yet sufficiently frequent in its occurrence for instances occasionally to fall under the notice of every practitioner of medicine.

2d. It is a remarkably latent affection, so far as concerns the symptomatic events usually distinguished as *vital* or *rational,* its existence, on the other hand, being determined with great ease and positiveness by physical signs. It is therefore very often overlooked, and confounded with other affections.

3d. Although as yet inadequately studied by means of analytical investigations, enough has been thereby developed, of late, to show that the notions pertaining to the pathology, progress and treatment of the disease, which are commonly entertained, and inculcated in standard treatises, are, in some important particulars, erroneous.

4th. As a reason wholly personal, the writer would add, that having a short time since analyzed a collection of the cases observed and recorded by himself,* he has on that account felt greater interest in the examination of fresh clinical illustrations of the affection.

---

Case I.—*Chronic, succeeding acute Pleurisy. Improvement sufficient to return to labor after five months. Relapse after continuing at labor five months. Case coming under observation fifteen months from date of acute attack. Renewed inflammation, a second time, while progressing favorably, with superadded bronchitis. Case still under observation.*

Wm. Thompson, aged 32, groom, unmarried, Irish, father lately living and well, and supposes his mother also to be alive. Has six sisters and one brother living, and has lost one sister from the effects of an injury. October 10, 1853.

*Previous History.*—Prior to about 15 months ago he enjoyed excellent health. Attacked suddenly at night, after exposure on the same night to cold and wet, with sharp, lancinating pain in the right side, felt especially with the act of inspiration. He was confined to the bed about three months, was bled twice copiously, blistered and salivated. In about five months he had recovered sufficiently to return to labor, and continued to labor for five months, during which period he says that he enjoyed excellent health. At the end of this five months, about five months prior to this date, (Oct. 10,) he was attacked with chills and treated for intermittent fever. Was confined to the bed for about three weeks. Had no pain in the chest at that time, and has had none there since. The chills were repeated every alternate day for a week. He has not felt able to labor since, and entered the hospital a month ago.

*Present Symptoms.*—Up and dressed, able to walk about and render some assistance in the ward. Aspect pallid; prolabia exsanguine; appetite tolerable; perspires freely at night, but this has been the case for a short time only; bowels regular; urine tolerably abundant under the use of diuretics

---

* Essay on Chronic Pleurisy, based on an Analysis of Forty-seven cases. By Austin Flint, M. D., 8vo., pp. 56.

which he has taken for some time; slight cough, but no expectoration; skin-cool; hands somewhat congested; pulse exceedingly small and feeble, ninety six per minute; tongue pallid and thinly coated; respirations twenty-four.

PHYSICAL SIGNS.

*Inspection.*—Right shoulder depressed, and upper part of right side flattened. Intercostal depressions visible at the lower lateral porrion of right side. Slight breathing movement on this side, in this respect contrasting strongly with the left side. A considerable lateral curvature of spine, the convexity looking to the left side.

*Mensuration.*—Right side, seventeen and one-eighth inches; left side, sixteen and seven-eighths, (spinal curvature to be borne in mind.)

*Percussion.*—Left side yields very clear vesicular resonance.—Right side, above the third rib, gives a feeble resonance, tympanitic in quality. Below the third rib, flatness, except on strong percussion, when an obscure tympanitic sound is developed.

*Palpation.*—Vocal fremitus at the summit of the chest, behind, on the right side, and none on left side in corresponding situation. In front, same situation, strong fremitus on right, and feeble on left side. Over middle and lower thirds, fremitus on left, and none on right side.

*Succussion.*—On grasping the shoulder of each side and throwing them backward, the sternal extremity of the right clavicle is observed to be movable, contrasting in this respect strikingly with that of the left side.

*Auscultation.*—At summit of chest, on right side, shortened inspiration, a brief interval, and a prolonged expiration, the latter more intense, and higher in pitch than the sound of inspiration. On left side, marked, exaggerated, vesicular respiration. A full distant, respiratory sound appreciable over middle third of right side in front. Behind, over scapula, very feeble respiration, too feeble to note character, and no respiratory sound below the scapula. No râles on either side. Vocal resonance below spine of scapula, about equal on both sides, but much more marked on the right side above the spinous ridge. In front, present on both sides, but much more marked on the right side; apex impulse of the heart not discoverable.

The previous history in this case, shows that the patient was attacked with acute pleurisy fifteen months before the foregoing record was made. The chronic affection, in this instance, succeeded the acute, which is not the rule, although it is so stated in systematic works.* The patient, from his account, was treated with active remedies, which, however, did not prevent his being confined to the bed for three months, and rendered unable to labor for five months. And, now, what was the condition of the chest during the following five months that he was able to labor, and considered himself in good health? Considerable progress had doubtless been made in the resorption of the liquid effusion which the chest must previously have contained, but it is probable that the pleural sac still contained liquid. It is not surprising that, under such circumstances, the patient should suppose the recovery to be complete, for cases are met with, in which the chest is filled on one side, and not enough inconvenience experienced to prevent laborious occupations.† In all cases that recover, the evidences of re-established health are such that patients deem themselves entirely well, long before physical explorations determines the restorative processes of absorption and adhesion to have been completed. Always many months, and sometimes even years elapse before recovery from the local affection is fully accomplished. During this time, without due precaution, the patient is constantly in danger of renewed inflammatory action. Imprudence in returning to labor, and, in all probability, in other respects, led to this result in the present instance; and the patient, with a large re-accumulation of liquid effusion within the chest, at the date of the foregoing record, was apparently a second time progressing slowly toward recovery.

In this case it is to be remarked, that, so far as the rational, or vital symptoms are concerned, the evidences of chronic pleurisy were very slight. Putting aside the acute, lancinating pain at the date of the first attack, fifteen months before, there were few symptoms, past or present, denoting that the chest was the seat of a grave affection, and none characteristic of the particular affection under which the patient was laboring. There had been no pain since cough was light; the respiration but little increased in frequency, and, in fact, the case had been regarded, before his admission, as solely one of intermittent fever. Without the advantage of physical exploration, a positive diagnosis would have been almost impossible. A correct diagnosis is seldom made in these cases by practitioners who do not avail themselves of the assistance afforded by physical exploration. The disease is called consumption,

* *Vide* Essay by writer. † *Vide* Essay by writer.

intermittent fever, dyspepsia, general debility, and often goes by the title of that ideal affection *liver complaint.*

To determine the character of the disease with the aid of physical signs is one of the easiest problems in physical diagnosis. In the great majority of instances the signs, already given as present in this case, are found in combination, viz:—enlargement of the side affected, at first, determined by inspection and measurement, and contraction afterward; diminished breathing movement of the side affected, approaching sometimes to immobility, and a corresponding increase of mobility on the unaffected side; flatness of the percussion sound from the base of the chest, extending upward in proportion to the quantity of liquid, and diminished resonance above the level of the liquid; the bronchial respiration at the summit of the chest over the site of the condensed lung, with, frequently, vocal resonance and fremitus due to the condensation, and on the unaffected side, if the lung be free from disease, clearness of vesicular resonance on percussion, and an exaggerated vesicular respiration.

Other signs of minor importance will be noticed in connection with the other cases.

With several signs thus associated, all of which are appreciated without difficulty, a failure to recognize a well marked case of chronic pleurisy can hardly occur to one tolerably conversant with the principles and practice of physical exploration.

---

Dec. 6.—This patient continued to report progressive improvement up to about a week prior to this date. Since the former record, Oct. 10, he has been up and dressed daily, and has acted as an assistant to the ward attendant, performing considerable labor. During this interval his cough was slight, with little or no expectoration. About a week ago, he was attacked, in the night time, with severe cough, and in a day or two began to expectorate. The expectoration was at first small, and gradually became abundant. From the commencement of this new attack he has had pain in the chest, which he refers to the lower half of the sternum, extending laterally in both directions. Up to this attack his appetite had been good, but he has since had little desire for food. Is thirsty, especially at night. Bowels open daily, or on alternate days. He sweats profusely every night. Dr. Dickinson, the resident physician, has observed febrile movement for a week past, especially at evening. Tongue, on the morning of this date, moist and clean. Skin cool and mellow. Pulse ninety-two and soft.

### PHYSICAL SIGNS.

*Inspection.*—Right side does not present marked contraction any where. The two sides appear to be not far from equal in volume. The right side is nearly immovable, while the left presents increased expansive and elevation movements in respiration. The intercostal spaces on the left side, at the inferior lateral portion of the chest, are depressed, and no depressions are visible on the right side.

*Percussion.*—Over left side, at superior anterior portion, very clear vesicular resonance. The right side is extremely tender, so that moderately forcible percussion gives great pain. Flatness exists universally in front and behind, save in infra clavicular space, there is a distinct but faint tympanitic resonance.

*Auscultation.*—Notably exaggerated vesicular respiration on the left side, the inspiration long and expansive, with a sound of expiration short and lower in pitch than that of inspiration. On right side, in front and behind, no distinctly appreciable sound of respiration. Heart sounds very audible. Vocal resonance on the left side, marked in front and behind, more especially in the infra clavicular region. None on right side, save in the infra clavicular and scapular regions, and here relatively feeble. Vocal fremitus on left side, marked everywhere, especially posteriorly below scapula. On right side entirely absent, save in infra clavicular region, and here more marked than in corresponding region of left side. Heart impulse not felt, but visible between fourth and fifth ribs, directly beneath nipple.

---

In the interval between the dates of the records, above quoted the patient was apparently progressing favorably. He was gaining in strength, improving in aspect, and, in fact, considered himself to be in tolerable health. During this period he had no active treatment directed specially to the chest affection. It was intentional, and not from neglect, that this course was pursued, the observation of previous cases having satisfied the writer that after the measures designed to promote resorption of the liquid effusion, viz:—mercurialization, diuretics, hydragogue cathartics, vesication, etc., have been continued for a short period, a few weeks usually, they have accomplished all that is to be expected from them, and to push them farther tends to retard if not to prevent recovery. It is generally practicable, by more or less of the measures just mentioned, employed in combination, or succession, to produce, within a short space of time, a marked diminution in the quantity of the effused liquid; but having reached a certain point, this diminution ceases to go on with rapidity, and the patient thereafter suffers from the general effects of the remedies, while the great object for which they are employed is no longer attained. Before coming under my charge the patient had received treatment directed to the chest affection, having been a month in the hospital, and the plan now adopted was to endeavor to improve the constitutional vigor. For this end tonic remedies were directed, a little porter and nutritious diet. In several instances I have witnessed the steady progress of the disease toward recovery under this plan of management, after all special efforts, by way of medication, to hasten the removal of the liquid effusion were abandoned. These efforts, under judicious limitations, are useful. They will generally effect a certain amount of utility, the amount varying in different cases; and having reached this limit they are no longer useful, but, if persisted in, will do harm. This seems to me a highly important practical point in the management of chronic pleurisy. How is it to be determined when they have ceased to be useful? Mainly by the evidence afforded by physical exploration. When the physical signs show that appreciable progress in the work of resorption no longer follows the operation of the remedies, they are to be abandoned, and the leading indication for treatment then is to develop and sustain the power of the constitution, and thus indirectly, but still efficiently, promote the processes of restoration. This is stated as the result of clinical observation. If we were to seek an explanation, it is to be found, probably, in the fact that, the morbid contents of the pleural sac, consisting of febrin and turbid serum, the serous or watery portion alone is made to enter the vessels by endosmosis as the result of diminishing the specific gravity of the blood by diuretics, cathartics, blisters, etc. The reduction in the quantity of the liquid effusion effected by these remedies ceases, so soon as the resorption raises the specific gravity of the effusion which remains to a point too high for the operations of the law of endosmosis. After this point is reached, the liquid is taken up very slowly, as the febrin becomes separated, solidified and organized.

At the last date, the patient was evidently suffering from a renewal of pleuritic inflammation, accompanied by a bronchitis.—The physical signs, compared with those recorded Oct. 10, show an increase in the liquid contents of the chest. There was ground for the suspicion that a purulent conversion had taken place, rendering the case one of empyema, instead of the ordinary form of chronic pleurisy. Whether this conversion has taken place, is a clinical question often not easily decided by the symptoms and signs. The occurrence of chills and hectic paroxyms is presumptive evidence, but not reliable, since they are observed in cases of simple chronic pleurisy. Stronger evidence is afforded by the quantity of liquid not being reduced, or continuing to increase notwithstanding the use of remedies designed to produce resorption, as shown by the physical signs, and especially, according to the opinion of some observers, by a marked distension of the intercostal spaces. The organization of pus, as is well known, offers a physical obstacle to endosmosis, greater than is presented when the effused liquid consists of serum with more or less coagulated fibrin. The propriety of the operation of paracentesis was considered, in this case, under the present circumstances, but as the patient was laboring under a recent acute attack, and, although the quantity of fluid in the right pleural sac was large, no difficulty of respiration being experienced, nor any evidences of defective hematosis manifested, it was concluded, with the advice of Dr. Dudley, attending surgeon to the hospital, to postpone it.

---

Dec. 18.—Patient reports greatly improved. He his up and dressed, and his aspect is much better than on the preceding date. The cough is less troublesome, and the quantity of expectoration diminished. The right side now appears again moderately contracted. The intercostal depressions on this side are again visible. Feeble respiration is heard over the right scapula, and in front on the right side, above the third rib. The tenderness has much diminished. The amount of effusion has evidently diminished since the 6th inst. The patient is in a fair way to recover from the effects of the late attack.

This is the condition of the patient at the time of writing. The case will continue under observation, and the question of resorting to paracentesis is, for the present, deferred. Statistics show that in a large proportion of cases of chronic pleurisy, recovery, in the end, takes place under judicious management, and with the observance of due precautions. Under all the circumstances, notwithstanding the duration of the affection, a favorable prognosis may probably be entertained in this case, and in the absence, as yet, of a sufficient number of observations showing the results of paracentesis in similar instances, there is certainly room for doubt whether the chances of recovery would be increased at the present moment by the operation, when it is considered that there is not embarrassment of the respiratory function sufficient to occasion immediate danger, or, indeed, any suffering.

A favorable prognosis in this, and similar cases, of course involves the absence of tuberculous disease. It is a current opinion that chronic pleurisy is very frequently associated with tubercle, either as a sequel or an antecedent affection. This opinion will be found in most modern works which treat of chronic pleurisy.—The results of numerical analysis, however, show that it is very far from the truth, the connection with tubercle existing in only a small proportion of instances.* In the present case, and in the other cases remaining to be noticed in this paper, the evidences of tubercule are wanting up to the time that the patients remained under observation. How is the co-existence or non-existence of tubercle to be determined? The condition of the lung on the side in which the pleuritic effusion exists, precludes the application of physical exploration, to ascertain whether it contains a tuberculous deposit or not. So far as physical signs are concerned, the question is to be settled by an examination of the side not affected with pleurisy. Recollecting the law of tuberculosis, that the deposit takes place first at the apex of one lung, and shortly afterward at the apex of the other lung, if we find the non-pleuritic side entirely free from all signs of disease, it follows either that the lung on the pleuritic side is not the seat of the deposit, or that, if there be any deposit, it has taken place too recently for the opposite lung to have become affected. Various symptoms will assist in arriving at a conclusion with respect to this point. If the disease be complicated with tubercle, cough and expectoration will be likely to be more or less prominent, the latter being solid, or purulent. These symptoms, it is true, may be present without tubercle. They do not alone prove the existence of the latter, but only increase the probabilities of this being the case; while, on the other hand, the absence of cough and a solid or purulent expectoration, is strong, but not conclusive proof against the co-existence of tubercle. In many, if not most cases of simple chronic pleurisy, cough and expectoration are either absent, or are not prominent symptoms. Notable and progressive emaciation should create suspicion of tubercle, patients affected with pleurisy alone, frequently preserving considerable *embonpoint.* Hemoptysis must also be deemed an ominous symptom, but in one of the cases that have fallen under my observation, hemorrhage occurred repeatedly, without other indications of the existence of phthisis, complete recovery taking place, the patient at the present time enjoying excellent health.

---

* *Vide* Essay.

CASE II.—*Disease subacute from the beginning. Case received into a hospital as one of ship fever. Patient performing duties of day laborer after six weeks, and continuing so employed for nearty three months. Attacked with intermittent fever, and entered the hospital to be cured of that affection. Pleuritic inflammation not renewed. Discharged from the hospital after about four weeks, reporting quite well, the chest still containing considerable liquid effusion.*

ANDREW MAHER, aged twenty; laborer; been in America five months; father, mother, three brothers and one sister in Ireland, living and well when last heard from; admitted Oct. 25.

The patient stated, on his admission, that he had always had good health prior to coming to this country, but was attacked with ship fever shortly after his arrival, and was ill for a month. He then came to Louisville, and was employed as a laborer on the Jeffersonville railroad up to the time when he dated the commencement of his present illness. Two weeks before his admission he was attacked with intermittent fever, the paroxysms recurring daily for four days, when they were arrested by the sulphate of quinia, but again returned every alternate day, and continued up to the time of his admission. During the two weeks prior to his admission, he had repeated attacks of epistaxis, by which he thinks he lost some quarts of blood.

At the time of his admission his aspect was notably anemic. There were present anorexia, nausea and vomiting, thirst, pulse one hundred and fourteen. The day previous he had had a chill followed by febrile movement. He stated that he had not at this, or any previous period had cough, or other pulmonary symptoms. No examination of the chest was made at this time. The disease was supposed to be simply intermittent fever, and the sulphate of quinia was prescribed in dose of gr. xx. in the twenty-four hours.

---

Oct. 27.—Patient has had no recurrence of paroxysms of intermittent fever since his admission. Reports better. Complains only of debility. Stated again, that he had no cough or expectoration. Pulse ninety-six. Perspired freely the previous night. Keeps the bed from sense of debility.

*Physical Signs.*—The breathing movement is notably greater on the left than on the right side. The left side appears larger in volume than the right.

Dullness on percussion exists on the right side as low as the interspace between the fourth and fifth ribs, and flatness below the fifth rib. Over the left side clear vesicular resonance. Over the left side, in front, exaggerated vesicular respiration with an expiratory sound lower in pitch than that of inspiration. On the right side, in front, the respiratory sound tolerably evolved, but notably less in degree than on the left side; the vesicular quality less marked; the inspiration higher in pitch. Vocal resonance about equal on both sides. Vocal fremitus somewhat greater on the right side at the summit of the chest. Right semi-circumference, in line of nipple, sixteen and one quarter inches, the left, sixteen and three quarter inches, making the contraction of the right side an inch.* Right interscapular space, measured a little above the inferior angle of scapula, half an inch less on the right, than on the left side. No spinal curvature.

*Treatment.*—The citrate of iron and sulphate of quinia, and nutritious diet.

On asking the patient if he has ever had pain in the right side, he replies in the affirmative. He states that he had pain in that situation after being attacked with what was called ship fever, shortly after his arrival in this country. It appears, that being taken ill soon after landing, he entered a hospital, and was there told that his disease was ship fever. He remained in the hospital a week only. He was considered well enough to be discharged after the lapse of a week. He had been confined to the bed for two days only, before entering the hospital. He had no pain in the chest till after his discharge from the hospital. No physical exploration of the chest was made while in the hospital. The pain was situated at the inferior anterior and lateral portion of the right side. It was not constant. He describes it as sharp, and felt especially in inspiration. He did not work for three weeks after leaving the hospital. States that he was too weak to work, and he noticed, also, that on active exercise he was put out of breath. When he commenced to work, three weeks after leaving the hospital, he found that he could not do his accustomed amount of labor. He was wanting in breath, and the pain in the right side returned. He was obliged to give up work after continuing the attempt for six days, and he then got a blister for the right side. He rested a week, and then again went to work. He was now able to work pretty well, and he continued at labor till he was attacked with intermittent fever, two weeks before his admission to the Louisville hospital. In the mean time. (a period of about three months) he had no pain in his

---

* Assuming the normal semi-circumference of the right side to be half an inch greater than that of the left side.

side, and was not sensible of being put out of breath by exercise more than usual.

---

This case differs from that previously given, in the fact, that the pleuritic inflammation was subacute at the commencement; in other words, the affection did not succeed acute pleurisy. In this respect the case accords with the larger number of the cases of chronic pleurisy met with in practice.

The case illustrates, in a striking manner, the latency of the disease, as regards the rational or vital symptoms, and the liability, without resorting to physical exploration, to overlook it, or confound it with other affections. The patient after an illness of two days, has recourse to a hospital. He presents no prominent ailments referrible to the chest, and, being an immigrant just arrived, it is presumed that he labors under typhus fever. That this was not the fact is sufficiently shown by his being well enough to be discharged from the hospital, after remaining a week. After leaving the hospital, as he states when questioned with reference to the point, he had some lancinating pains in the right side.—This circumstance he had forgotten, or deemed it not of sufficient importance to mention of his own accord. He was unable to labor for three weeks after leaving the hospital, in consequence of debility and want of breath. At this time the chest doubtless contained an abundant liquid effusion. He attempts to labor after three weeks, but after a trial of six days, is obliged to give it up from pain in the side and want of breath; but after resting another week, he makes a second attempt with better success, and finds himself able to do his accustomed amount of labor continuously for nearly three months, when he is attacked with intermittent fever from which he suffers for two weeks, and then comes to the hospital. In this case again is exemplified the fact that with considerable, and even a great amount of effusion within the chest, patients are capable of performing hard manual labor, with the usual exposure, etc., for it is not to be supposed that five weeks after the commencement of the disease much progress had been made in absorption, since the chest contained considerable liquid five months afterward, at the time the foregoing record was made. The process of absorption was slowly going on, probably, during the time he was constantly employed as a day laborer, and unconscious of any important malady.

The case shows, also, that although persons thus situated are exposed to renewed attacks of inflammation, as was illustrated in the first case, yet that they may escape under circumstances when *a priori* this could hardly have been expected. There was no renewal of the inflammation in consequence of the muscular exertions, exposure, and imprudences of various kinds incident to the life of an Irish rail-road laborer. Nor did an attack of intermittent fever, lasting two weeks, reproduce the pleuritis; for, when the patient entered the hospital, and during his stay there, no evidences were present of an affection of the chest, except that the processes of restoration from chronic pleurisy were not completed.

---

Nov. 8.—This patient was able to sit up, a day or two after the date of the previous record, and has constantly improved up to this date. He is now employed as an assistant to the ward attendant. His aspect is much improved. He reports quite well, has no pain, and is not put out of breath on exercise. Appetite is good, and bowels regular. A careful physical examination, with more minuteness of detail, made on this date, led to the following results:

*Inspection.*—Right side contracted at the lower third. Breathing movement (expansive and elevation) notably greatest on the left side. This disparity especially marked on forced respiration. The contraction apparent behind, as well as in front. The right shoulder depressed. No curvature of spine. Right nipple about half an inch lower than the left.

*Mensuration.*—Width of inter-scapular space on left side, three inches; on right side, two and three-quarter inches, at upper part. Near lower angle of scapula, on right side, two and three-quarter inches, on left side three and one-quarter inches. Semi-circumference of right side through the nipple, sixteen and one-quarter inches; semi-circumference of left side, sixteen and three-quarter inches.

*Percussion.*—At summit of right chest, above third rib, notable dullness; over and below third rib, dullness, approximating to flatness; below interspace between fourth and fifth ribs, positive flatness. Marked dullness in right supra and infra spinous spaces behind; dullness still greater over lower portion of scapula, and flatness below the inferior angle.

*Auscultation.*—At summit, in front, on left side, well evolved vesicular murmur with inspiration, and no sound of expiration appreciable. On right side very feeble, almost inappreciable sound of inspiration, with a rather feeble but distinct and prolonged sound of expiration. Absence of al respiratory sound over the middle and lower thirds, on right side. Behind, over right scapula, a very feeble sound of inspiration. Absence of respiratory sound over lower part of right scapula and below scapula. On left side, exaggerated vesicular respiration. Bronchophony at summit of right, and not on left side. Absence of vocal resonance over middle and lower thirds of right side. Behind, moderate vocal resonance over the left scapula, but more marked over the right; none below scapula on either side.

Vocal fremitus, marked at summit of right side, and not on left; none on either side over middle and lower thirds.

*Width of Intercostal spaces.*—Between sixth and seventh ribs on right side, on vertical line through nipple, distance one inch. On left side, on corresponding line, one and a quarter inch.

The patient continues to report absence of cough and expectoration. The treatment with citrate of iron, and the sulphate of quinia, and nutritious diet is continued.

---

Nov. 20.—Patient reports quite well and wishes to be discharged. A physical examination made on this date showed some increase in the measurement of the chest, on both sides, the patient having evidently gained in weight since he entered the hospital. The dullness on percussion above the fourth rib on the right side had diminished. In other respects the signs remained the same.

The patient was discharged on this date, the right chest, as appears from the examination just referred to, still containing considerable liquid effusion.

---

Case III.—*Disease having existed for several years, its precise duration not determinable, owing to absence of pain, and other symptoms denoting Pleuritic Inflammation. Patient able to do hard labor while laboring under the disease. Progress toward recovery notwithstanding drunkenness, etc. Patient still in hospital.*

David Laten, aged 42, laborer, admitted Nov. 16.

From the account given by the patient at the time of his admission, he appeared to be laboring under intermittent fever, and was placed under treatment for that affection, Shortly after his admission he had an attack of delirium tremens and recovered in a few days. He then began to complain of some cough and pain in the right side.

A physical examination of the chest was not made till the 13th of December. In the mean time, the patient had seemed to be improving, was up and dressed, and about the ward, reporting daily that he was better. The existence of some cough, pain in the right side, and a pallid aspect led to the suspicion of some pulmonary affection.

### PHYSICAL SIGNS.

*Inspection.*—Right shoulder somewhat depressed. The inferior part of right chest appears contracted. The summit of right chest relatively flattened. Breathing movement of right side less than of the left.

*Mensuration.*—Right semi-circumference, just below the nipple seventeen inches; left, seventeen inches. Right interscapular space at lower angle of scapula, three and three-quarter inches, left, five inches. Patient in sitting posture with arms evenly folded.

*Percussion.*—Behind, relatively (to left side) dull over whole right side. Dullness more marked over scapula than below. In front, dullness above lower margin of third rib, and flatness below this point. Flatness over the whole axillary region. Clear vesicular resonance over the left side.

*Auscultation.*—In front, at the summit of left side, well evolved vesicular respiration, low in pitch, inspiratory sound alone heard. At the summit of the right side in front, inspiratory sound alone heard, higher in pitch than on the left side, and less vesicular. Disparity in these points between the two sides marked. Below third rib, on the right side, nothing heard but the heart sounds. Behind, over right scapula, inspiration alone heard, feeble, vesicular, and rather high in pitch. Below scapula, inspiration better evolved and vesicular. On left side, respiratory characters concealed by a sibilant râle, heard both at the upper and lower portions of the chest. Vocal resonance more marked at summit of right, than of left side in front. None on either side in front over middle and lower thirds; behind, slight over right scapula, and none over the left; more marked below right scapula, and none below the left. Vocal fremitus marked at the summit of the right side, in front, and none on the left; none over middle and lower thirds on either side; slight over right side, behind, below scapula, and none on the left side.

Heart impulse very feeble between the fifth and sixth ribs, on vertical line with nipple. Heart sounds pure. Tenderness on pressure over lower portion of right side.

The account of the previous history given by the patient is as follows:

He has suffered from intermittent fever, more or less, for several years past, especially during the autumn and winter. Has never been laid up with an acute affection accompanied by pain in the chest. He does not recollect ever having experienced pain in the right side, but has formerly had some pain in the left side! Since entering the hospital, however, has felt some pain in the right side. Has had some cough with expectoration for several years. Has at the present time about the same as usual. Has observed that his breath has been short on exercise for several years, but he has not experienced much difficulty from this source. Has worked as a laborer constantly during the summer months, but during the winter has generally worked but little. Has been able to do a good day's work in the summer. Has never had hemoptysis. His aspect is pallid. He is not emaciated, and has kept at about the same weight for several years. He is habitually in the use of ardent spirits, and addicted to excesses in their use. Respiration sixteen. Pulse sixty-six.

The patient still remains in the hospital, the foregoing examination having been made but a few days prior to the time of writing this record.

---

This case is interesting from the fact that the patient has doubtless labored under chronic pleurisy, or an accumulation of liquid effusion within the chest, in consequence of that disease, for a long time, probably for several years, but it is impossible, from his account, to determine at what date it commenced. The patient cannot recall any local symptoms significant of a pleuritic inflammation at a former period. He has been conscious of not being well for several years, but he has attributed his ill health entirely to repeated attacks of intermittent fever, nor has he ever been told that he was laboring under any other affection. The physical signs show a certain amount of fibrin and liquid in the right pleural cavity, but the contraction of the chest, in this, as in the preceding case, is evidence that the quantity of liquid effusion has been greater at a former period, a considerable portion having been removed by absorption. The processes of restoration have thus been going on, notwithstanding his performing the duties of a laborer in the summer season, the frequent occurrence of intermittent fever, and his being an habitual drunkard. That the disease, under such unfavorable circumstances has not proved fatal, illustrates its intrinsic tendency to recovery.

In this, as in the first case, is exemplified the small amount of inconvenience to which a considerable collection of liquid in the pleural cavity of one side may give rise. The patient has been able to do hard labor during the summer season without difficulty, although he has noticed that his breath was somewhat deficient on active exercise.

It is very evident that in such a case, without the aid of physical exploration, it would have been impossible to have divined the nature of the pulmonary affection. No suspicion of chronic pleurisy was entertained during the time he remained in the hospital before the chest was examined. Hence it is that so often cases of this disease are called consumption, dyspepsia, general debility, *liver complaint*, etc.

The reader will not fail to remark, that in this, and the preceding case, the favorable tendency of the disease is shown, not only by the progress toward recovery in spite of unfavorable influences, but by the fact, that the patient had received no medical treatment directed specially to the chest affection, the latter not having been determined. The ratio of recoveries of uncomplicated ordinary chronic pleurisy under medical treatment is large;* and when we see, in cases like these, the processes of restoration go on, without treatment, and under most untoward circumstances, the question arises, whether the proportion of recoveries may not always depend more on an intrinsic tendency of the disease, than upon the efficacy of the therapeutical measures pursued. This question would perhaps be forced upon us oftener than it is, were it practicable to obtain statistics showing the progress and rate of mortality of different diseases uninfluenced by medication. As the true point of departure for estimating the efficacy which belongs to therapeutics, such statistics are as desirable as they are difficult to be obtained.

---

CASE IV.—*Disease of five weeks' duration. Considerable progress made toward recovery, without medical treatment; the patient laboring under intermittent fever. Case remaining under observation.*

THOMAS BUTLER, aged 39, Irish laborer, admitted December 8th.

*Previous History.*—Patient states that he enjoyed uninterrupted good health prior to three years and four months ago. He then began to suffer from attacks of intermittent fever. He had frequent relapses of this disease, but no other affection that he knows of, until during the last winter

*See Essay by [illegible]ter.

(the precise period he cannot state,) he had a lancinating pain in the right side, situated laterally at about the eighth rib. The pain was acute on taking a deep inspiration, and on coughing. He was not at this time confined to the bed, nor to the house, but walked a mile to obtain medical advice, after having had more or less of the pain for eight days. The physician whom he consulted applied a blister over the seat of the pain, after which the pain shifted its seat and became more diffused, but in three days it again was concentrated in the particular site above mentioned when another blister was applied, immediately after which the pain disappeared, and did not again return. The two blisters were the only remedies employed. He went to work two days after the last blister was applied, and he continued to work, being in good health, till about a month afterward he had a relapse of intermittent fever. He states that during the month between the occurrence of the pain and the relapse of intermittent fever, he had no pain, no cough, nor difficulty in breathing, and was as well as at any period in his life. He has continued to have relapses of intermittent fever, arresting the paroxysms with remedies, up to the present time. About two months ago, while working in a stone quarry, he was attacked with pain, commencing at about the third lumbar vertebra, and shooting downward into the right hip. This pain was not constant but occurred in paroxysms. He suffered from this pain, more or less, for two weeks, but it did not oblige him to quit working. After this pain ceased to trouble him, he felt perfectly well, and, in the mean time, became an ostler, and, as he says, after getting his feet wet, was attacked with a chill followed by febrile movement and sweating. About this time, (about five weeks ago,) he began to have pain in the left side. The pain was not severe. He also began to have some cough at about the same time, and the act of coughing occasioned pain over the whole left side. He took quinia directly after the paroxysms just mentioned, and had no recurrence at regular intervals, but had paroxysms occurring irregularly several times during the last five weeks. His cough has been troublesome, occurring in spells, and accompanied with slight expectoration. He has felt want of breath on exercise, and has been unable to work for the last five weeks.

PHYSICAL SIGNS.

*Present Symptoms.*—Aspect rather pallid; appetite capricious; tongue clear, but pale; bowels regular; occasional cough, with small expectoration; pulse seventy-two; respiration twenty-one.

*Inspection.*—Breathing movement, (expansive and elevation,) in a marked degree greater on the right side. The left side evidently contracted. The disparity in mobility and size apparent both in front and behind. The shoulders on a level. The intercostal depressions less marked on the left than on the right side. The left nipple higher than the right. No curvature of spine.

*Mensuration.*—Semi-circumference of right side, seventeen and one-half inches; of left side, sixteen inches. Width of right interscapular space at lower angle of scapula, three and one-half inches; of left, two and seven-eighths inches.

*Percussion.*—Marked relative dullness over the whole of left side, in front and behind. At the inferior portion of the chest the dullness amounts to flatness. Percussion sound over right side clear and vesicular.

*Auscultation.*—Respiratory sound on right side, vesicular, normal, tolerably evolved, not exaggerated. On left side, in front, at upper and middle thirds, inspiratory sound inappreciable, with a feeble, high pitched sound of expiration. At lower third, no respiratory sound. Behind, respiratory sound scarcely appreciable any where on left side.

*Vocal Resonance.*—Generally more marked over the right than over the left side. In the left axillary region more marked than in the right, and, also, in the left infra scapular region.

*Vocal Fremitus.*—More marked over the right side, in front and behind, save in the infra scapular region, where it is greater on the left side.

Apex impulse of the heart quite feeble, between the fourth and fifth ribs, directly beneath the nipple.

---

The previous history in this case renders it probable that the pleurisy occurred about five weeks before the date of the admission into the hospital. The pains which occurred several months before, situated in the right side, were undoubtedly neuralgic, as well as the pains in the loins and right hip, which preceded the attack of pleurisy. That neuralgic affections should have occurred in a patient suffering for three years, and more, from frequently repeated attacks of intermittent fever, is not surprising. In the five weeks from the date of the present disease, there has been an accumulation of a considerable quantity of liquid effusion within the chest, and considerable progress made in its removal by absorption. Both these facts are proved by the present contraction of the chest. The degree of contraction, under these circumstances, shows that the quantity of liquid has diminished, and is in a measure proportionable to the previous enlargement of the chest. That in so great a period, enlargement and subsequent contraction should have taken place; in other words, that the evidences of there having been a pretty large accumulation, and of the removal by absorption of a considerable portion, should be present so soon after the date of the attack, illustrates what is probably the rule in chronic pleurisy, viz: that the effusion takes place rapidly, the chest becoming more or less filled in a brief period from the date of the attack. The commencement of absorption may be indefinitely postponed, or, at all events, this is very variable in different cases. In this instance the process of absorption must have begun soon after the effusion took place. And here, again, is another instance in which considerable progress was made toward recovery without any aid from therapeutics. The patient had no medical treatment prior to his entering the hospital, nor had he taken any remedies. He had, probably, not had the advantage of the most favorable hygienic conditions, and moreover, had suffered from recurring paroxysms of intermittent fever. The intrinsic tendency of the disease toward recovery is exemplified, in a striking manner, by this case.*

---

The object of the writer in reporting the foregoing cases of chronic pleurisy, which, within the space of a few weeks have been the subjects of clinical remarks at the hospital amphitheatre, has been, as the reader will have perceived, to present, with brief comments, the illustrations which they afford of some of the interesting and important points pertaining to the development, progress, etc., of this disease, and of the physical signs, by means of which the diagnosis is to be made. In view of the fact, that a positive diagnosis cannot be made without, and is easily made with the aid of physical exploration, it will, perhaps, prove a convenience to some who may possibly have become interested by the foregoing cases and remarks, to give a *resume* of the physical signs, by which the practitioner who has given no previous attention to the subject may readily determine the nature of the affection.

* Dec. 24.— This patient eloped from the hospital shortly after the date of the last record.

PHYSICAL SIGNS ASSOCIATED IN CHRONIC PLEURISY.

1. *Signs determinable by Inspection.*—These are divisible in two classes, viz:—*First*, signs prior to absorption. *Second*, signs after the quantity of effusion has been more or less diminished by absorption.

Of the *first* class, the signs are as follows:—Enlargement of the affected side; diminished expansive and elevation movements in respiration, amounting sometimes to complete immobility; abolition of intercostal depressions, or bulging, i. e. elevated above the level of the ribs; interspaces between the ribs increased; obliquity in direction of ribs diminished.

Of the *second* class: visible contraction of the affected side; depression of the shoulder on that side; breathing movements still, relatively to the other side, lessened; intercostal depressions apparent, and abnormal approximation of the ribs; frequently lateral curvature of the spine.

On the healthy side, expansion somewhat increased, and the breathing movements greater than natural. When the quantity of effusion is very large, more or less œdema is usually present on the affected side, and the subcutaneous veins are more prominent than on the healthy side.

2. *Signs determinable by Mensuration.*—*First*, before absorption, increase of semi-circumference of affected side compared with that of the other side, if the quantity of liquid effusion be great; and the enlargement proportionable to the quantity.

*Second*, after absorption, diminished semi-circumference, proportionate to the progress made in absorption. Approximation of posterior margin of scapula to the spinal column.

3. *Signs determinable by Percussion.*—Flatness over the whole of the affected side, if the quantity of effusion be very large, and flatness from base of chest upward, according to the extent to which the liquid accumulates at the bottom of the pleural sac, and compresses the lung, pushing it upward and backward. Above the point to which flatness extends, dullness on percussion, compared with the other side, due to condensation of the lung substance by compression.

The boundary line dividing the inferior *flat* portion of the chest, from the superior *dull* portion, is well defined, the transition being abrupt, and well marked. Occasionally this line of demarkation will be found to vary with the position of the patient, being lower down when the patient is recumbent, than when in a standing or sitting posture. In a large proportion, however, in cases of chronic pleurisy, the latter test of the presence of liquid effusion is unavailable.

Occasionally a tympanitic resonance, more or less obscure, on the affected side. When situated below the level of the liquid, this must be transmitted sound due to gas in stomach or intestines. Above that point, it may be still transmitted, or due to gas in the pleural cavity, or possibly, to air in the pulmonary vesicles. On the healthy side, the percussion sound unusually clear. Sense of resistance greater, in other words, less opposition, within the chest, to acting, by pressure, or percussion, on the elasticity of the ribs, on the affected, than on the healthy side.

4. *Signs determinable by Auscultation.*—Over as much of the affected side, from the base upward, as there is flatness of the percussion sound, absence of respiratory sound, the heart sounds being alone heard. Occasional exceptions to this rule, which, it is stated, are more apt to occur in children. Above the line of flatness, a respiratory sound generally appreciable, but very variable in intensity in different cases, presenting more or less of the modifications proper to the *bronchial respiration*, in other words, having more or less completely the characters of the normal bronchial respiration heard in many persons at the sterno-clavicular junction in front, and at the upper part of the interscapular space behind, and, also, in an exaggerated degree, over the trachea. These abnormal modifications, or characters, are the following:—the quality of sound, more or less tubular, or blowing, in other words, the vesicular quality characteristic of normal pulmonary respiration lessened, or absent; the inspiration shortened, and higher in pitch than the normal pulmonary respiratory sound; an interval between the sounds of inspiration and expiration, if a sound of expiration be present; a prolonged expiration, if it be present, having a higher pitch than the sound of inspiration.

The vocal resonance generally more marked at the summit of the affected side over the condensed lung, than over the corresponding situation on the healthy side. Occasional exceptions to this rule, see No. 4, of the foregoing cases. On the healthy side, the respiratory sound, if the chronic pleurisy be uncomplicated with tubercle, or other affection of the lungs, presenting the normal characters of the vesicular murmur, often exaggerated. Under these circumstances the increased intensity, without other change, of the murmur, very properly has suggested the title, *supplementary respiration.**

---

*I do not enumerate under the head of the auscultatory signs, friction sounds, and the vocal sign called egophony. The former are rarely heard in cases of chronic

5. *Signs determinable by Palpation.*—More or less tenderness on pressure of the affected side. Vocal fremitus at the summit of the affected side, over the condensed lung, usually more marked than over the same situation on the healthy side. Occasional exceptions to this rule, see case, No. 4. Below the level of the liquid effusion absence of all fremitus, and, if the patient naturally have a fremitus, there will be a contrast presented by its presence on the healthy side.

The apex impulse of the heart frequently removed from its normal position. In pleurisy of the right side with large effusion, before absorption, the heart usually pushed in a direction upward and to the left; after absorption, returning, is sometimes drawn to the right. In pleurisy of the left side, removed in a direction to the right, and upward, carried beneath the sternum and occasionally even beyond the right margin of the sternum. In some instances the apex impulse not perceptible, nor visible, and the abnormal situation of the heart only determinable by ascertaining at what point the sounds are heard with a maximum of intensity.

6. *Signs determined by Succussion.*—A greater mobility of the clavicle on the affected side was observed in one of the cases reported. (No. 1.) No sounds are developed by this mode of exploration.

pleurisy, according to my experience; and the latter still more unfrequently. Moreover, it appears to be settled that egophony when sufficiently distinct is not pathognomic of the presence of liquid effusion.

